# Altered immune status of circulating T lymphocytes during sepsis: children also

**DOI:** 10.1186/s13054-014-0486-0

**Published:** 2014-08-14

**Authors:** Jean-Marc Cavaillon

**Affiliations:** Unité Cytokines & Inflammation, Institut Pasteur, 28 Rue Dr. Roux, 75014 Paris, France

## Abstract

Altered immune status of blood leukocytes is a general phenomenon observed in adult patients with sepsis or septic shock. This is also the case in children with septic shock for both T helper 1 and T helper 2 lymphocytes, as demonstrated by their reduced *ex vivo* cytokine production upon activation by phytohemagglutinin.

In a recent paper published in *Critical Care*, Muszynski and colleagues [1] report that, in children with septic shock, purified blood CD4+ T lymphocytes display an altered responsiveness to a non-specific mitogen (phytohemagglutinin A (PHA)) as assessed by reduced production of IL-2, interferon (IFN)-γ, IL-4 and IL-10. They also showed that lipopolysaccharide (LPS)-induced TNF is decreased, and that the percentage of circulating regulatory T lymphocytes (Tregs) is unchanged.

This is probably the first study establishing that in children, as in adults, the immune status of circulating CD4+ T lymphocytes is altered in terms of cytokine production. The early demonstration of an altered T-cell response in adult patients with sepsis was reported in 1977, when a significant reduced delayed-type hypersensitivity to recall antigens was observed [2]. Unfortunately, other functional tests to precisely assay the adaptive responsiveness of T cells (for example, response to antigens, specific cytotoxicity, and so on) are scarce.

Different observations in Muszynski and colleagues’ study are of interest. First, the authors report that the mortality was only 9%, a value far below that regularly reported for adults with septic shock [3]. This low percentage of deceased children is in agreement with the known low mortality rates in children for several infectious and non-infectious diseases compared with adults [4]. This may reflect that children would be less prone to develop a severe cytokine storm since they display lower capacity to release cytokines [5]. Indeed, the levels of plasma cytokines found by the authors appear lower than those usually reported in adult patients with septic shock.

Second, another very interesting observation is that all PHA-induced cytokines were reduced in children with septic shock and persistent or nosocomial infection, independent of whether they were classified as T helper (Th)1 (IFNγ, IL-2) or Th2 (IL-4, IL-10) cytokines. This is against the dogma claiming that sepsis is associated with an up-regulation of the Th2 response and a down-regulation of the Th1 response. However, this fits with a previous study in adults with sepsis or non-infectious systemic inflammatory response syndrome (SIRS) [6], and with another report in trauma patients [7] in whom production of both *ex vivo* Th1 and Th2 cytokines was altered. Furthermore, the authors showed that the decreased capacity of CD4+ T lymphocytes to produce cytokines was transient and normal responsiveness was restored by day 7. A similar observation was made for LPS-induced TNF production, which was restored by day 7 except in the two non-surviving children. This is reminiscent of what was already reported for cytokine production by LPS-activated monocytes in adults with sepsis, which remained below normal levels in non-surviving patients [8].

It is worth mentioning that circulating lymphocytes have been shown not to be the main leukocyte population with altered gene profiles during septic shock. Indeed, in pediatric septic shock, Wong and colleagues [9] showed that 109 genes were up- or down-regulated in all types of lymphocytes, whereas 965 genes in monocytes, and 2,360 genes in neutrophils were modulated. Whether this observation reflects enhanced apoptosis of lymphocytes in sepsis compared with other leukocytes remains to be fully examined. Great differences in transcriptomic response according to developmental age were noted in children with septic shock [10]. Because of the small number of children included in the study, the authors could not determine whether the observed alteration was identical in neonates, infant and school-age children.

The authors made their observation using PHA; surprisingly, in a study performed in adult sepsis a similar observation could not be achieved with this mitogen but only with concanavalin A [6]. This difference may be explained by different parameters of the experimental procedure (length of incubation, whole blood versus purified T-cell subset, addition of fetal bovine proteins, and so on). Because PHA response is due to a T-cell subset [11], and is mainly dependent on accessory cells [12,13], one cannot exclude that the observation also partially reflects an altered cooperative role of accessory cells (monocytes, dendritic cells). The median percentage purity of CD4+ cells was 93%. Since as little as 0.03% adherent cells are sufficient to allow PHA-induced proliferation by purified T cells [14], a sufficient number of accessory cells were still present to allow the PHA-induced cytokine production. Anyhow, other authors who employed anti-CD3 plus anti-CD28 coated beads or plates to bypass the need for accessory cells and to target all CD4+ and CD8+ T lymphocytes made similar conclusions regarding the immune status of T lymphocytes in adult patients with sepsis [15,16].

In addition, the authors analyzed the presence of circulating Treg cells, and found an unchanged percentage. In this context it is worth mentioning that, at homeostasis, a balance exists between IL-2 producer cells and Tregs, which sepsis most probably disrupts [17]. In contrast, in adult sepsis the percentage of circulating Tregs is enhanced but their absolute number is roughly unchanged compared with healthy volunteers as a reflection of the global lymphopenia [18]. Finally, and most importantly, it is worth recalling that the immune status of T lymphocytes in tissues might be completely different to that revealed by the analysis performed in the blood stream after sepsis or sterile SIRS [19,20].

## Abbreviations

IFN: Interferon
IL: Interleukin
LPS: Lipopolysaccharide
PHA: Phytohemagglutinin A
SIRS: Systemic inflammatory response syndrome
Th: T helper cell
TNF: Tumor necrosis factor
Treg: Regulatory T lymphocyte
